# Abacavir pharmacokinetics in African children living with HIV: A pooled analysis describing the effects of age, malnutrition and common concomitant medications

**DOI:** 10.1111/bcp.14984

**Published:** 2021-08-12

**Authors:** Tjokosela Tikiso, Helen McIlleron, David Burger, Diana Gibb, Helena Rabie, Janice Lee, Marc Lallemant, Mark F. Cotton, Moherndran Archary, Stefanie Hennig, Paolo Denti

**Affiliations:** ^1^ Division of Clinical Pharmacology, Department of Medicine University of Cape Town Cape Town South Africa; ^2^ Wellcome Centre for Infectious Diseases Research in Africa (CIDRI‐Africa), Institute of Infectious Disease and Molecular Medicine University of Cape Town Cape Town South Africa; ^3^ Department of Pharmacy Radboud University Medical Centre Nijmegen the Netherlands; ^4^ MRC Clinical Trials Unit at University College London London UK; ^5^ Department of Paediatrics and Child Health and Family Centre for Research with Ubuntu (FAM‐CRU) Stellenbosch University and Tygerberg Children's Hospital Cape Town South Africa; ^6^ Drugs for Neglected Diseases initiative Geneva Switzerland; ^7^ Department of Paediatrics and Child Health at King Edward VIII Hospital affiliated to the Nelson R Mandela School of Medicine University of KwaZulu‐Natal South Africa; ^8^ Certara, Inc. Princeton New Jersey USA; ^9^ School of Clinical Sciences, Faculty of Health Queensland University of Technology Brisbane QLD Australia

**Keywords:** abacavir, children, efavirenz, lopinavir, malnutrition, population pharmacokinetics, rifampicin

## Abstract

**Aims:**

Abacavir is part of WHO‐recommended regimens to treat HIV in children under 15 years of age. In a pooled analysis across four studies, we describe abacavir population pharmacokinetics to investigate the influence of age, concomitant medications, malnutrition and formulation.

**Methods:**

A total of 230 HIV‐infected African children were included, with median (range) age of 2.1 (0.1–12.8) years and weight of 9.8 (2.5–30.0) kg. The population pharmacokinetics of abacavir was described using nonlinear mixed‐effects modelling.

**Results:**

Abacavir pharmacokinetics was best described by a two‐compartment model with first‐order elimination, and absorption described by transit compartments. Clearance was predicted around 54% of its mature value at birth and 90% at 10 months. The estimated typical clearance at steady state was 10.7 L/h in a child weighing 9.8 kg co‐treated with lopinavir/ritonavir, and was 12% higher in children receiving efavirenz. During coadministration of rifampicin‐based antituberculosis treatment and super‐boosted lopinavir in a 1:1 ratio with ritonavir, abacavir exposure decreased by 29.4%. Malnourished children living with HIV had higher abacavir exposure initially, but this effect waned with nutritional rehabilitation. An additional 18.4% reduction in clearance after the first abacavir dose was described, suggesting induction of clearance with time on lopinavir/ritonavir‐based therapy. Finally, absorption of the fixed dose combination tablet was 24% slower than the abacavir liquid formulation.

**Conclusion:**

In this pooled analysis we found that children on lopinavir/ritonavir or efavirenz had similar abacavir exposures, while concomitant TB treatment and super‐boosted lopinavir gave significantly reduced abacavir concentrations.

**What is already known about this subject**

- The pharmacokinetics (PK) of abacavir have been characterized previously in children; however, the effects of growth and development, drug–drug interactions and other covariates on abacavir PK remain limited.

**What this study adds**

- There was a decrease in abacavir exposure when coadministered with LPV/r, LPV/r plus rifampicin, efavirenz.
- Malnourished children had high and variable exposures that normalized as nutritional status resolved.
- Abacavir exposures in children on the recommended 8 mg/kg twice daily or 16 mg/kg once daily dosing are comparable to the adult exposures, as well as exposures seen in other studies conducted in children.

## 1 INTRODUCTION

In 2018, 84 000 children from eastern and southern Africa acquired HIV.^1^ Approximately 940 000 of 1.7 million children under 15 years living with HIV were receiving combination antiretroviral treatment (ART) in 2018. The World Health Organization (WHO) recommends two nucleoside reverse transcriptase inhibitors (NRTIs), abacavir and lamivudine, in first‐line ART for children younger than 15 years. In children younger than 3 years, the third component is lopinavir co‐formulated with ritonavir (LPV/r in a 4:1 ratio). In children older than 3 years of age, the non‐nucleoside reverse transcriptase inhibitor (NNRTI) efavirenz, which was previously recommended, has recently been replaced by dolutegravir.^2^, ^3^ HIV is often complicated by associated conditions such as malnutrition and co‐infections. Despite its wide use in children, knowledge of abacavir pharmacokinetics is limited, with scant information to guide improved dosing in children of various age groups in the context of malnutrition, drug–drug interactions and other covariates.

Abacavir is indicated for children over 3 months of age at 8 mg/kg twice daily or 16 mg/kg once daily.^4^, ^5^ Abacavir is extensively metabolized by the liver, with less than 2% excreted unchanged in urine.^6^ The two major pathways of abacavir metabolism involve alcohol dehydrogenase (ADH) and uridine diphosphate glucuronyltransferase (UGT) enzymes, producing inactive carboxylate and glucuronide metabolites.^6^ Previous studies report that coadministration with food and formulation has no effect on abacavir exposure.^7^, ^8^ However, abacavir solution has been associated with an 11% higher peak serum concentration (*C*_max_) than the tablet formulation.^9^ Its binding to plasma proteins is about 50%.^7^

Many physiological systems are not functioning optimally in children with malnutrition. Total body water is increased, plasma albumin is decreased, Phase I and II metabolic reactions are considerably reduced as the severity of malnutrition increases.^10^, ^11^, ^12^ These physiological alterations may either directly or indirectly influence the pharmacokinetics of abacavir.^13^

Drug–drug interactions among antiretrovirals and anti‐tuberculosis drugs are common. When adults on abacavir are coadministered LPV/r, abacavir exposure decreases by approximately 30%.^14^ In patients co‐treated for TB with a rifampicin‐based regimen, lopinavir would decrease by up to 90% with the standard LPV/r 4:1 dosing, so additional ritonavir is administered to achieve a 4:4 ratio with lopinavir (super‐boosted lopinavir) to counteract the interaction. We previously showed that abacavir exposure is reduced by 36% when children were co‐treated with rifampicin and super‐boosted lopinavir.^15^ Cohort studies in children raised concern that some abacavir‐containing regimens may be less effective than regimens with a different nucleoside reverse transcriptase inhibitor backbone.^16^, ^17^ Drug–drug interactions with companion NNRTIs or protease inhibitors (PIs) resulting in reduced abacavir exposures could potentially contribute to such findings.

The purpose of this pharmacokinetic meta‐analysis was therefore to pool several available abacavir clinical datasets to take advantage of the increased sample size and perform a more robust analysis to investigate the consequence of differences in body size, age, concomitant TB co‐medications, malnutrition and drug formulation on abacavir pharmacokinetics in children.

## 2 METHODS

### 2.1 Clinical studies and data

This pooled analysis used data from ARROW (Uganda and Zimbabwe),^18^ CHAPAS‐3 (Uganda and Zambia),^19^ DNDi (South Africa)^20^ and MATCH (South Africa).^21^ Briefly, the objects in regards to PK of the three individual studies were: in ARROW, to compare the pharmacokinetics of once daily *vs* twice daily dosing of abacavir and lamivudine when given together with nevirapine or efavirenz; in CHAPAS‐3, to compare abacavir, stavudine or zidovudine as dual‐ or triple fixed‐dose combination paediatric tablets with lamivudine and nevirapine or efavirenz; in DNDi, to test whether adding extra ritonavir to co‐formulated lopinavir/ritonavir (4:1) would overcome the effect of rifampicin on lopinavir exposures; and in MATCH, to describe the pharmacokinetics of antiretrovirals in paediatric patients with severe acute malnutrition as defined by the WHO. In all the studies, abacavir was administered orally following WHO weight‐band dosing, which targets an average dose (within each weight band) of 8 mg/kg twice daily or 16 mg/kg once daily. The sample profiles were intensively sampled on separate visits, the detailed distribution of patients and their characteristics across study visits are provided in Table 1. Of the 230 children available for analysis, in 227 children, pharmacokinetic sampling was performed during twice daily dosing. Forty‐one of these children also underwent pharmacokinetic evaluation while receiving once daily doses, while three children received daily doses only. A total of 154 children were on concomitant lopinavir/ritonavir and 76 were on efavirenz. Rifampicin‐containing anti‐TB treatment was administered to 104 children; of these, 101 were on super‐boosted lopinavir/ritonavir (4:4) and three on efavirenz. There were 115 malnourished children, characterized in this analysis as having weight‐for‐age and height‐for‐age *Z*‐score less than −2.0. The majority of children in our analysis received abacavir with LPV/r (4:1) and therefore were the reference group in the model.

**TABLE 1:** Distribution of patients and their characteristics across study visits in the analysis

	Participants (samples)	Weight (kg)	Age (years)	Weight‐for‐age Z‐score^a^	TB co‐infected (*n*)^b^
**ARROW**					
VISIT 1 (ABC + EFV) BID	39 (272)	19.5 (14.0; 29.5)	7.4 (4.0; 12.5)	−1.15 (−3.23; 0.54)	0
VISIT 2 (ABC + EFV) OD	41 (326)	20.5 (14.0; 29.5)	7.7 (4.1; 12.6)	−1.12 (−3.01; 0.20)	0
**CHAPAS‐3**					
VISIT 1 (ABC + EFV) BID	24 (180)	15.7 (10.7; 27.9)	4.7 (2.1; 12.8)	−1.04 (−3.94; 1.15)	3
VISIT 2 (ABC + EFV) OD	3 (24)	14.4 (13.2; 19.7)	3.8 (3.3; 4.7)	−0.83 (−0.97; 0.78)	
**DNDi**					
VISIT 1 (ABC + LPV/r + RIF)	85 (497)	8.8 (3.9; 14.9)	1.6 (0.3; 5.3)	−1.93 (−5.19; 1.39)	85
VISIT 2 (ABC + LPV/r + RIF)	74 (436)	9.5 (4.9; 15.9)	1.9 (0.3; 5.7)	−1.38 (−4.84; 1.56)	74
VISIT 3 (ABC + LPV/r)	71 (405)	10.0 (6.8; 15.9)	2.1 (0.8; 5.8)	−1.39 (−4.86; 1.51)	0
**MATCH**					
VISIT 1, delay in initiation of ART (ABC + LPV/r)	34 (135)	7.5 (2.6; 11.7)	1.2 (0.2; 3.7)	−2.80 (−5.63; −0.37)	6
VISIT 2, delay in initiation of ART (ABC + LPV/r)	30 (146)	7.6 (3.3; 12.2)	1.4 (0.3; 3.2)	−2.05 (−4.71; 0.32)	6
VISIT 1, delay in initiation of ART (ABC + EFV)	5 (20)	12.0 (9.8; 19.0)	3.6 (3.4; 8.4)	−2.48 (−3.25; −2.13)	0
VISIT 2, delay in initiation of ART (ABC + EFV)	4 (19)	17.3 (11.2; 19.0)	7.8 (3.7; 8.5)	−2.58 (−2.73; −2.54)	0
VISIT 1, early initiation of ART (ABC + LPV/r)	33 (132)	6.2 (2.5; 17.0)	0.8 (0.2; 10.8)	−3.59 (−6.29; −1.03)	4
VISIT 2, early initiation of ART (ABC + LPV/r)	30 (145)	6.8 (3.3; 23.5)	0.9 (0.2; 10.9)	−3.31 (−6.29; 0.17)	7
VISIT 1, early initiation of ART (ABC + EFV)	2 (8)	16.6 (13.6; 19.6)	8.6 (6.5; 10.7)	−3.71 (−3.72; −3.69)	0
VISIT 2, early initiation of ART (ABC + EFV)	3 (15)	15.5 (14.5; 25.0)	9.9 (6.6; 10.7)	−3.89 (−4.55; −3.25)	0

The data are reported as median (range).

ABC, abacavir; BID, twice daily; EFV, efavirenz; LPV/r, lopinavir/ritonavir; OD, once daily; TB drugs, rifampicin‐based TB treatment.

PK in ARROW, CHAPAS‐3 and DNDi was taken at least 1 month after treatment start. Visit 2 in MATCH was on average after 14 days.

^a^ Z‐scores calculated according to WHO (<10 years) and CDC (>10 years) growth charts.

^b^ Additional ritonavir used for super‐boosting lopinavir during rifampicin‐based TB treatment.

### 2.2 Analytical methods

The analytical methods have previously been described in depth in the original published articles for each analysis. Plasma abacavir concentrations from DNDi, CHAPAS and MATCH studies were determined with a validated liquid chromatography–tandem mass spectrometry (LC–MS/MS) assay developed in the Division of Clinical Pharmacology, University of Cape Town. The lower limit of quantification (LLOQ) was 0.0243 μg/mL for DNDi, and 0.0238 μg/mL for CHAPAS and MATCH. The plasma concentration of abacavir from the ARROW study was determined using validated mass spectrometry and high‐performance liquid chromatography (HPLC) by GlaxoSmithKline (Research Triangle Park, NC, USA). The LLOQ for abacavir was 0.0243 μg/mL.

### 2.3 Population pharmacokinetic analysis

Data from each study were explored separately and added one by one starting from those with more intensive data, as suggested in Svensson et al.^22^ After the inclusion of each dataset, the model fit was reassessed and modified if necessary.

The population pharmacokinetics of abacavir was described using nonlinear mixed‐effects modelling (NONMEM 7.4.4) with auxiliary software (PsN, Pirana and Xpose), which were used for automation and diagnostics during the model‐building process.^23^ The first‐order conditional estimation with eta‐epsilon interaction (FOCE‐I) was used to estimate pharmacokinetic parameters.

Single‐ and multi‐compartment models with first‐order elimination and absorption (with or without an absorption lag time or transit compartments) were evaluated. Between‐subject (BSV), between‐visit (BVV) and between‐occasion variability (BOV) of random effects were tested on pharmacokinetics parameters and were assumed as lognormally distributed. Each dose was treated as a separate occasion, while consecutive evening and morning doses were grouped within the same visit. BVV and BSV were tested on clearance and volume of distribution, while BOV was tested on absorption parameters.^24^

The additive error for all samples was set to be at least 20% of the LLOQ of the assay, and the study specific LLOQ can be found in Table 2. Below the limit of quantification (BLQ) concentrations were handled with the M6 method as described by Beal.^25^ Briefly, the first BLQ value after the peak (or the last in a series of BLQ values before the peak) was imputed to half the lower limit of quantification (LLOQ/2) and included in the fit with their additive error inflated by LLOQ/2, while any subsequent BLQ values (or preceding if before the peak) were excluded from the fit and only considered for visual predictive check (VPC) diagnostics.

**TABLE 2:** Clinical characteristics of patients and demographics in studies included in the analysis

	Abacavir pooled analysis				
	ARROW^18^	CHAPAS‐3^19^	DNDi^20^	MATCH^21^	COMBINED
**Patients in analysis (*n*)**	41	27	87	75	230
**Males (*n*)**	17	13	38	41	109
**Samples in analysis (*n*)**	598	204	1338	620	2760
**Lower limit of quantification (μg/mL)**	0.0243	0.0238	0.0243	0.0238	–
**Age (years) median (IQR)**	7.6 (4.0–12.6)	4.7 (2.1–12.8)	1.9 (0.3–5.8)	1.4 (0.2–10.9)	2.1 (0.2–12.8)
**Age < 1 year (%)**	0	0	28.7	42.7	24.8
**Weight (kg) median (IQR)**	20.5 (14.0–30.0)	15.4 (10.7–27.9)	9.5 (3.9–15.9)	7.4 (2.5–25.0)	9.8 (2.5–30.0)
**Abacavir formulation**	FDC	FDC	Liquid	Liquid	–
**Concomitant medication (*n*)** ^**b**^					
**3TC + EFV**	41	24	0	8	73
**3TC + EFV + RIF**	0	3	0	0	3
**3TC + LPV/r (4:1)**	0	0	71	58	129
**3TC + LPV/r (4:4) + RIF**	0	0	87	15	101
**Weight‐for‐age Z‐score** ^a^ **median (IQR)**	−1.13 (−3.09–0.544)	−0.952 (−3.94–1.15)	−1.46 (−5.19–1.55)	−3.19 (−6.29–0.323)	−1.71 (−6.30–1.55)
**Malnourished (*n* (%))** ^**c**^	6 (14.6)	3 (11.1)	45 (51.7)	61 (81.3)	115

ABC, abacavir; EFV, efavirenz; 3TC, lamivudine; FDC, fixed‐dose combination; IQR, interquartile range; LPV/r (4:4), super‐boosted lopinavir/ritonavir; LPV/r (4:1), standard lopinavir/ritonavir; RIF, rifampicin.

^a^ *Z*‐scores calculated according to WHO (<10 years) and CDC (>10 years) growth charts.

^b^ Values reflect the numbers of children on the drugs at PK evaluation.

^c^ Weight‐for‐age and height‐for‐age *z*‐score <2.0.

Model building was guided by the drop in the objective function value (ΔOFV; proportional to −2 log‐likelihood), inspection of goodness‐of‐fit plots, VPC, biological plausibility and clinical relevance. A decrease in OFV of more than 3.84 between two nested models after the addition of one parameter was considered significant at *P* < .05.

### 2.4 Investigating factors that influence abacavir pharmacokinetics

Allometric scaling by total body weight was introduced on all clearance and volume parameters to account for the known effect of body size on pharmacokinetics with exponents fixed to 3/4 for elimination and intercompartmental clearance and 1 for volumes of distribution.^17^, ^26^, ^27^ Total body weight (TBW) and fat‐free mass (FFM)^28^ were evaluated as alternative size descriptors on both disposition parameters. To account for maturation, a sigmoidal function of postmenstrual age was used (Equation 1):

(1) $$\text{maturation}=\frac{\text{PMAG}E^{\gamma }}{\left(\text{PMAG}E_{50}^{\gamma }+\text{PMAG}E^{\gamma }\right)},$$

where *PMAGE* denotes postmenstrual age, *PMAGE*_50_ is the value of *PMAGE* at which 50% of the maturation is complete, and γ is a parameter determining the shape of the relationship. Since no information on the actual gestational age of the children was available, it was assumed to be 9 months.

After inclusion of weight and age in the model, additional covariates were screened based on inspection of parameter *vs* covariate plots and physiological plausibility and retained based on statistical significance at *P <* .01. To describe the time‐changing effect of malnutrition that resolves with days on nutritional supplementation, an exponential function was used (Equation 2):

(2) $$\text{Malnutrition}={\mathrm{MAL}}_{0}\cdot e^{-\frac{\lambda_{\mathrm{MAL}}\cdot \text{time}}{\text{logn}\left(2\right)}}$$

where *MAL*_0_ is the initial value of the malnutrition effect at day 0 (before start of supplementation), *λ*_*MAL*_ is the half‐life of the process (in days) and time is the duration of the nutritional supplementation treatment (in days). The precision of the final parameter estimates was evaluated by sampling importance resampling (SIR).^29^

### 2.5 Simulations

Using the parameter estimates from the final model, Monte Carlo simulations were performed to generate steady‐state abacavir AUC_0–12_ during co‐treatment with standard LPV/r (4:1), efavirenz or rifampicin plus super‐boosted lopinavir. A 12‐hourly dosing and a target dose of 8 mg/kg based on the WHO weight‐band guidelines (weights from 3–35.9 kg) were used to simulate exposure in 57 014 in silico patients weighing 3–35.9 kg. The age‐weight combinations were generated from a weight‐for‐age model developed based on values from children with TB and hence consistent with the population for whom the dosing guidelines are designed.^30^ All simulated exposures were compared to the recommended 12‐hour adult median AUC of 6.02 mg·h/L as suggested by the European Medicines Agency.^31^

## 3 RESULTS

### 3.1 Data summary

Four studies contributing 2760 plasma concentrations from 230 children living with HIV were used in this pooled analysis. Of these, 285 plasma concentrations (10.3%) were below the LLOQ of which most were drawn pre‐dose. The median (range) age and weight were 2.1 (0.1–12.8) years and 9.8 (2.5–30.0) kg, respectively. The detailed patient and study characteristics and their distributions in each study are presented in Table 2.

### 3.2 Population pharmacokinetics

**FIGURE 1:**
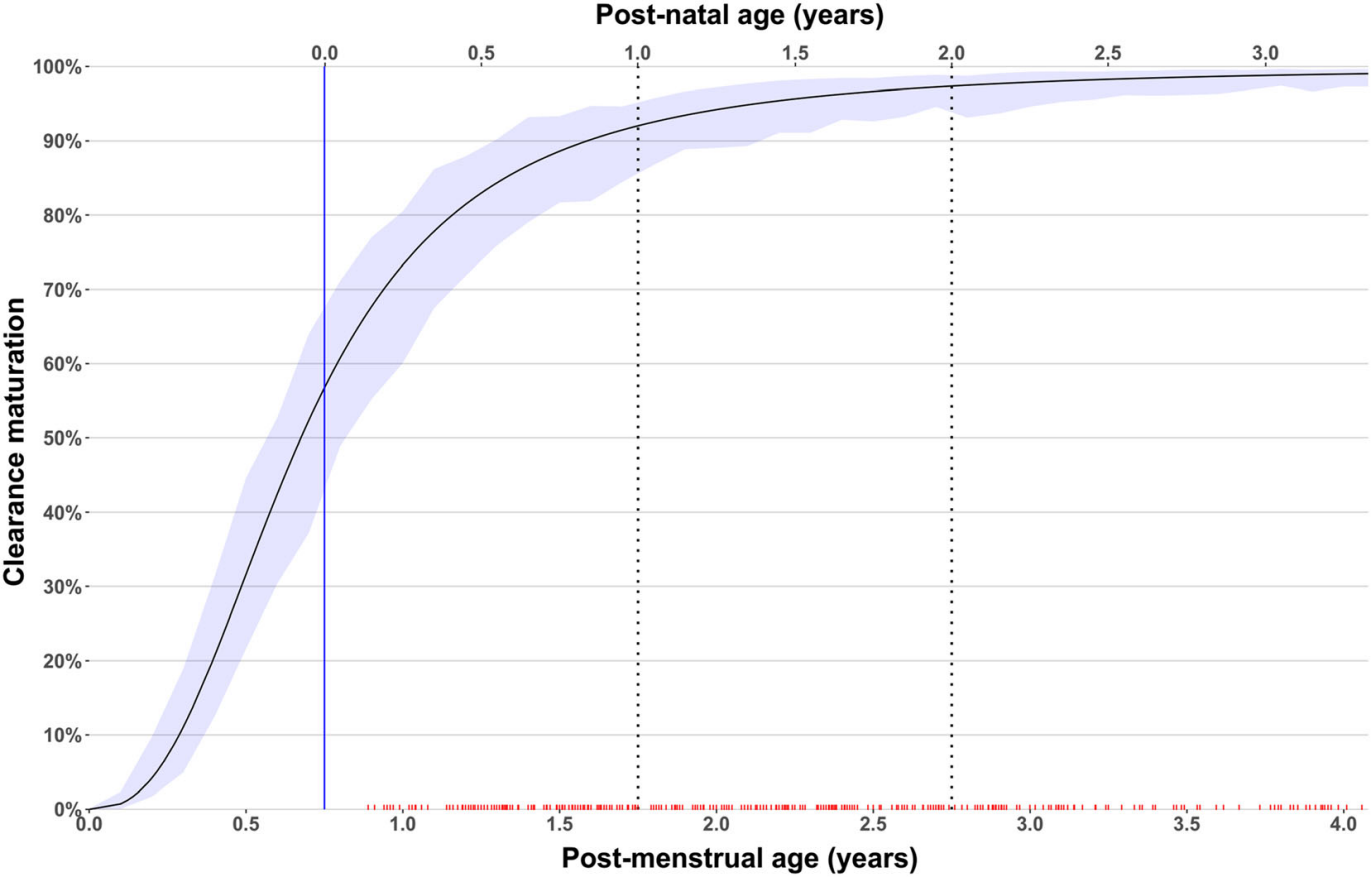
Maturation function of abacavir clearance *vs* post‐menstrual age (bottom x‐axis), or post‐natal age (top x‐axis, assuming average gestation of 9 months), after adjusting for weight. The shaded area represents the 90% confidence intervals. The solid vertical blue line represents birth, while the dashed vertical lines represent 1 year and 2 years post‐natal age respectively. The red ticks on the lower x‐axis represent the post‐menstrual age values available in our data

The population pharmacokinetics of abacavir was best described by a two‐compartment disposition model (difference in objective function value, ΔOFV = −728 when compared to a one‐compartment model, *P* < 10^−6^) with first‐order elimination and transit compartments describing absorption (ΔOFV = −148, *P* < 10^−6^, when compared with simple first‐order absorption). To adjust for differences in body size, allometric scaling of TBW was included for all disposition parameters and improved the model fit (ΔOFV = −268). Using FFM instead of TBW did not provide any significant improvements. After adjusting for body size, the effect of age on clearance was captured using a maturation function (ΔOFV = −15, *P* < 10^−3^). Clearance was predicted to be at 54% of its mature value at birth and 90% at 10 months. The maturation function of abacavir clearance with confidence intervals is shown in Figure 1. The apparent clearance (CL/F) for a typical 9.8 kg child co‐treated with standard LPV/r 4:1 at steady state was estimated at 10.7 L/h.

**FIGURE 2:**
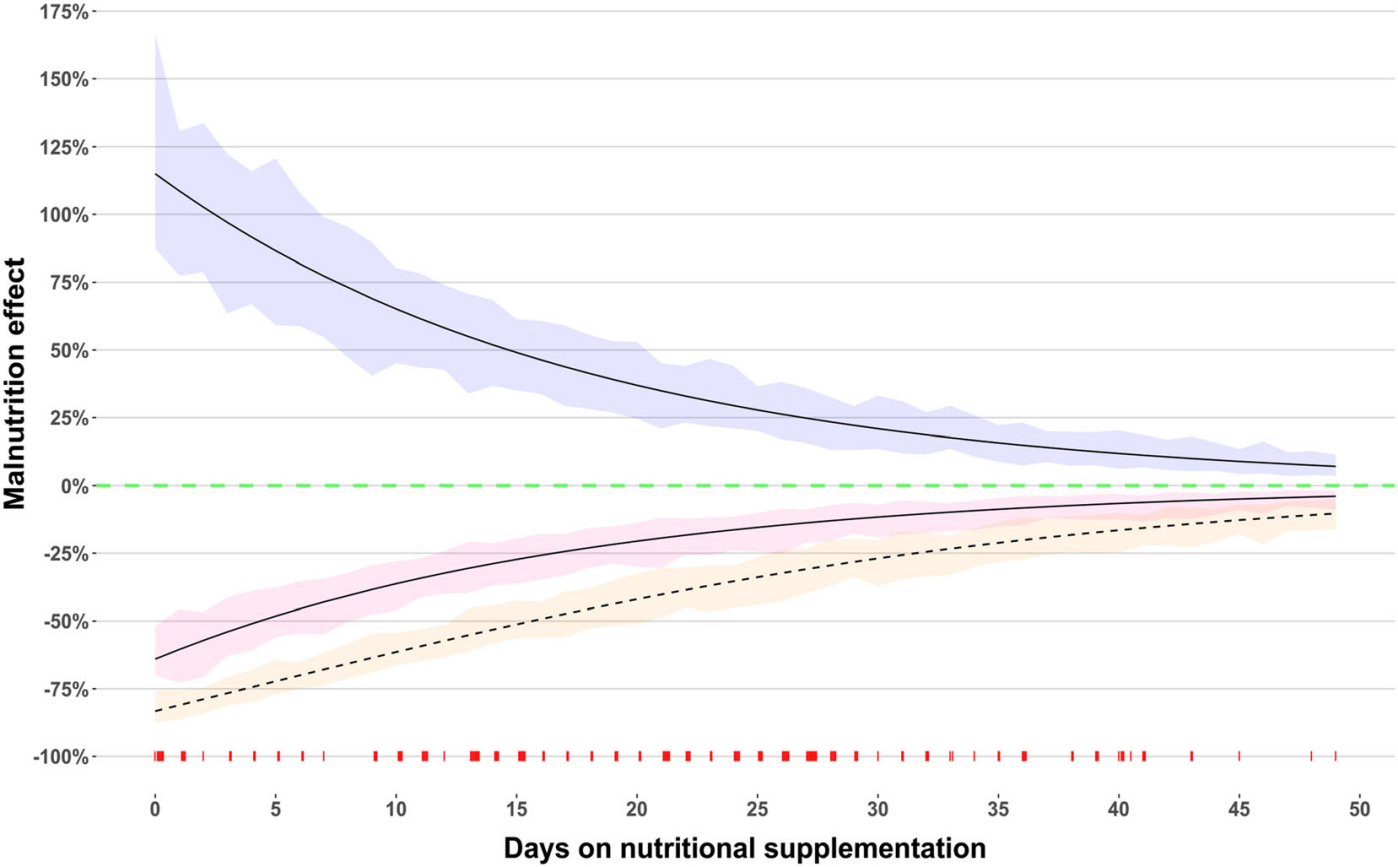
Effect of malnutrition on abacavir bioavailability (purple shaded area with a solid line), clearance (pink shaded area with a solid line) and clearance/bioavailability (yellow shaded area with a broken line) *vs* days on nutritional supplementation. The shaded areas represent the 90% confidence intervals. The y‐axis value of 0% (dotted green line) represents the values of a typical child co‐treated with standard LPV/r (4:1) at steady state after the resolution of malnutrition. The red ticks on the lower x‐axis represent the children available in our data on the days on nutritional supplementation

Clearance of the first abacavir dose was 18.4% lower (ΔOFV = −11.5, *P* < 10^−4^) than clearance for a typical child on standard LPV/r 4:1 for over 7 days. Coadministration with anti‐TB treatment plus super‐boosted lopinavir decreased abacavir bioavailability by 29.4% (ΔOFV = −48, *P* < 10^−6^). Decreased abacavir exposures was also seen in studies where abacavir was coadministered with protease inhibitors, as shown in Table 3. An increase in clearance of 12% was seen in children on efavirenz (ΔOFV = −10.9, *P* < 10^−4^). Malnourished children had higher and more variable exposures compared to a non‐malnourished typical child, as shown in Figure 2. The model captured this additional exposure by estimating that malnourished children have 115% higher bioavailability and 64% decreased clearance at the start of nutritional rehabilitation, but their exposure gradually normalized to that of the general population with a plasma half‐life of 12.2 days as their nutrition recovered (ΔOFV = −70.3, *P* < 10^−6^). At the start of nutritional rehabilitation, the compounded effect of reduced clearance and increased bioavailability produced an increase in AUC of more than 4‐fold, and the effect was still around 2‐fold after 14 days. Additionally, BOV on bioavailability (ΔOFV = −67, *P* < 10^−6^) and BOV on clearance (ΔOFV = −74, *P* < 10^−6^) was 1.39‐fold and 3.35‐fold larger for malnourished children compared to a typical child, respectively.

**TABLE 3:** Comparison of populations and AUCs between present pooled analysis and previous abacavir publications at steady state

Author	Study	Study arm	*n*	Location	Median weight [kg] (range)	Median age [yrs] (range)	Dose (mg/kg) per day	Formulation	Other ARVs	AUC_12	Original CL/F (L/h)	Allometry CL/F (L/h/ 70 kg^a^)
Pooled ABC	ARROW, CHAPAS, DNDi, MATCH	LPV/r (4:1)	230	South Africa, Uganda, Zambia, Zimbabwe	9.8 (2.5–30.0)	2 (0.2–12.8)	16	Liquid, tablet	3TC	10.2	10.7	46.8
		EFV								10.4	12.0	52.3
		LPV/r (4:4) + TB								6.66	7.55	33.2
		EFV + TB								7.58	12.0	52.3
Waters 2007		Control	24	UK	83.6	43.0 (31.0–62.0)	8	Tablet	3TC, ZDV	9.31	32.2	28.2
		ATV/r								7.57	39.6	34.7
		Control								7.57	39.6	34.7
		LPV/r (4:1)								5.24	57.3	50.2
Jackson 2012		Control	19	UK		45.0 (37.0–53.0)	8	Tablet	3TC, ZDV	6.77	44.3	38.8
		DRV/r								4.94	60.7	53.2
		RAL								6.99	43.0	37.6
Cella^b^ 2011	PENTA‐13	Children	70		23.8 (13.7–60.5)	5.9 (2.1–12.8)	16	Liquid, tablet	3TC, ZDV, NVP, EFV, LPV/r	6.96	33.2	74.6
	PENTA‐15	infants	70		12 (7.4–15.9)	1.8 (0.3–2.9)		Liquid		7.03	13.4	50.3
Zhao 2013	PENTA‐13 &15, ARROW	infants	21	Uganda, Zimbabwe	17.6 (7.6–60.9)	5.7 (0.4–12.8)	16	Liquid, tablet	3TC, ZDV, NVP, EFV, LPV/r	6.10	20.1	56.6
		Children	48							8.70		
Sleasman^b^ 2009	P1018	<18 years	15	USA	62.8 (37.6–89.2)	15.9 (13.7–17.6)	9.8	Tablet	3TC, ZDV, PIs, NNRTIs	7.01	42.8	46.4
		≥18 years	15		71.6 (44.1–122.7)	21.5 (18.5–24.3)	8.4			6.59	45.5	44.7
Jullien^c^ 2004			105	France	25 (2.5–84.0)	8.5 (0.1–16.0)	16	Liquid, tablet	NRTI+ PIs or NNRTI	8.5	23.7	51.3

ABC, abacavir; EFV, efavirenz; TB, rifampicin‐based TB treatment; LPV/r, lopinavir/ritonavir; 3TC, lamivudine; ZDV, zidovudine; ATV/r, atazanavir/ritonavir; DRV/r, darunavir/ritonavir; RAL, raltegravir; NVP, nevirapine; CL/F, apparent clearance; ARV, anti‐retroviral.

^a^ Original clearance values scaled to a 70 kg individual to allow for easier comparison of AUCs across studies.

^b^ Simulated patients were added to the original PENTA 13 (14 children) and PENTA 15 (23 infants).

^c^ Drug names for NRTI, NNRTIs and PIs not provided.

**FIGURE 3:**
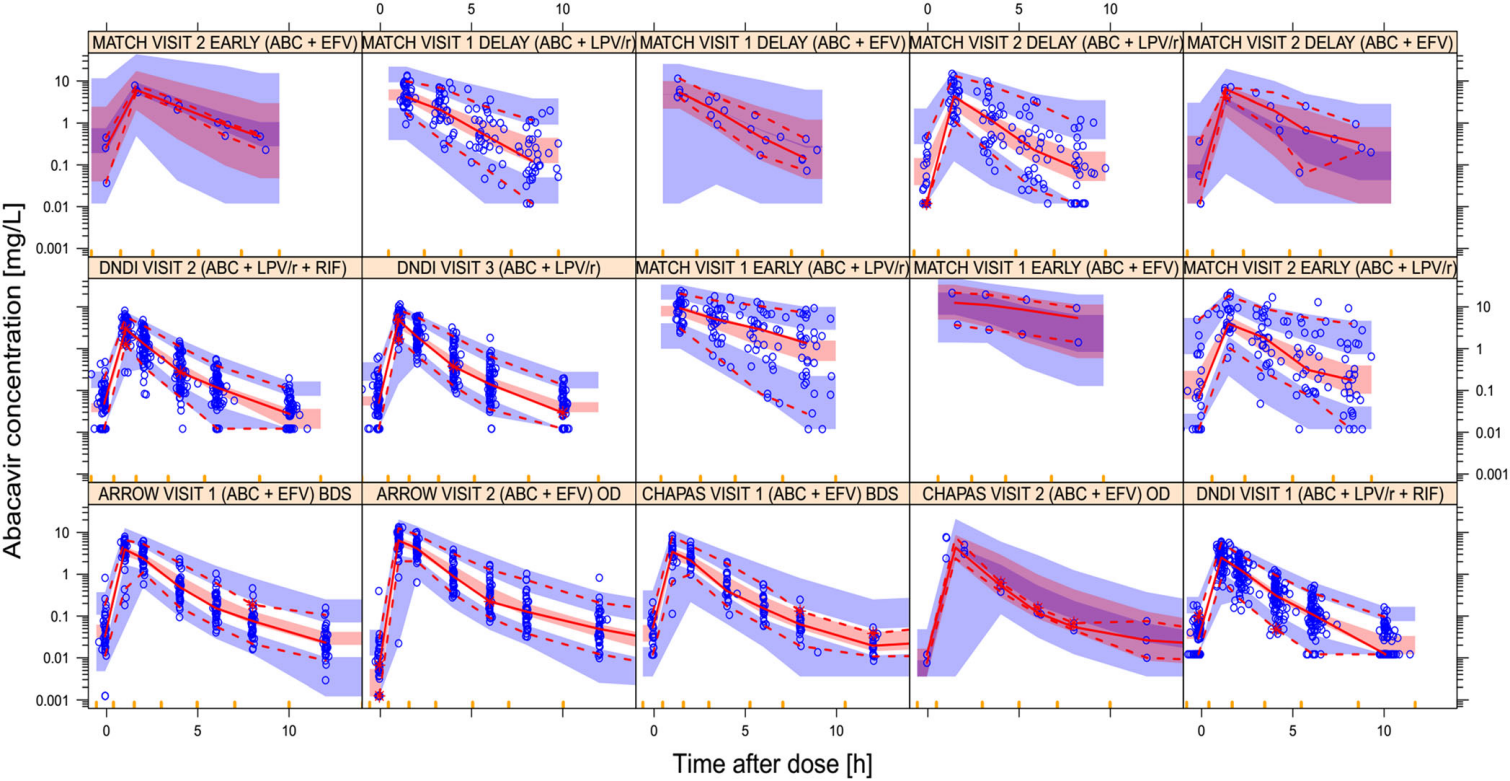
Visual predictive check (VPC) of abacavir concentration *vs* time after dose, stratified by study and PK visit. For an explanation of the meaning of each visit, please refer to Table 1. The solid and dashed lines represent the 5th, 50th and 95th percentiles of the observed data, while the shaded areas represent the model‐predicted 90% confidence intervals for the same percentiles. The dots are the observed concentrations. The yellow ticks are bin boundaries. The dots at the bottom of the VPC are BLQ value. ABC, abacavir; BLQ, below the limit of quantification; EFV, efavirenz; RIF, rifampicin; LPV/r, lopinavir/ritonavir; BDS, twice daily; OD, once daily

Abacavir plus lamivudine fixed‐dose combination tablets had a 24.9% slower absorption than abacavir liquid formulation (ΔOFV = −19, *P* < 10^−5^). For the children on twice‐daily dosing, the morning observed pre‐dose concentrations were often higher than the corresponding observed concentration at 8–10 hours after the morning dose administration. This was explained by an average delay of 2.52 hours in absorption for the evening dose, which led to significant model fit improvement (ΔOFV = −194, *P* < 10^−6^). The final parameter estimates with uncertainty are presented in Table 4 and a VPC stratified by study and visit is shown in Figure 3.

**TABLE 4:** Final parameter estimates with uncertainty for abacavir

Model parameter estimates	Typical value		Variability	
	Value	95% CI	% CV	95% CI
**Clearance (L/h) [CL]** ^a^	10.7	9.87; 11.5	14.5 (BSV)	11.7; 16.4
			15.3 (BVV)	13.3; 17.1
**Central volume of distribution (L)** ^**a**^	11.0	10.2; 11.7		
**First‐order absorption rate constant (1/h) [Ka]**	2.29	1.99; 2.56	77.3 (BOV)	69.1; 83.3
**Relative oral bioavailability [F]**	1 FIXED		39.2 (BOV)	35.1; 43.1
**Peripheral volume of distribution (L)** ^**a**^	3.33	2.97; 3.64	44.6 (BSV)	38.6; 49.4
**Inter‐compartmental clearance (L/h)** ^**a**^	1.10	0.97; 1.21		
**ƴ_maturation function** ^**b**^	2.57	1.84; 3.18		
**PMAGE**_**50**_^**b**^ **(months)**	8.10	6.30; 9.53		
**Mean absorption transit time (mins)**	6.24	4.96; 7.50	132 (BOV)	116; 145
**Number of absorption transit compartments**	11.9	8.66; 14.8		
**Proportional error (%)**	23.8	22.2; 25.0		
**Additive error (μg/L)**	2.01	1.51; 2.56		
**Change in F when on rifampicin + super‐boosted lopinavir**	−29.4	−35.8; −24.3		
**Change in CL for first abacavir dose (%)**	−18.4	−32.2; −7.50		
**Change in CL when on EFV (%)**	+12.0	+2.57; +20.1		
**Change in F of malnourished children at start of supplementation (%)**	+115	+67.4; +150		
**Change in CL of malnourished children at start of supplementation (%)**	−64.0	−75.4; −53.3		
**Malnutrition effect half‐life** ^**d**^ **(/day)**	12.2	−16.8; −9.87		
**Change in speed of absorption for fixed‐dose combination tablets (%)**	−24.9	−36.8; −17.2		
**Delay in absorption for night dose (h)**	2.52	2.10; 2.76		
**Extra BOV BIO in MATCH (fold change)**	1.39	1.13; 1.62		
**Extra BVV CL in MATCH (fold change)**	3.35	2.77; 3.85		

Between‐subject (BSV), ‐visit (BVV), and ‐occasion (BOV) variabilities were assumed as lognormally distributed and are reported as %CV (sqrt [omega]*100).

^**a**^ All clearances and volumes of distribution were allometrically scaled and the typical values reported here refer to a child weighing 9.8 kg on LPV/r (4:1) at steady state, the median value in the dataset.

^**b**^ PMAGE_50_ is the postmenstrual age at which 50% maturation is reached, while ƴ_maturation function is the shape factor in the sigmoidal maturation function.

^**c**^ The absorption mean transit time is the average time the drug spends travelling from the first transit compartment to the absorption compartment.

^**d**^ Malnutrition function denotes the amount of change in clearance and bioavailability per time.

### 3.3 Simulations

**FIGURE 4:**
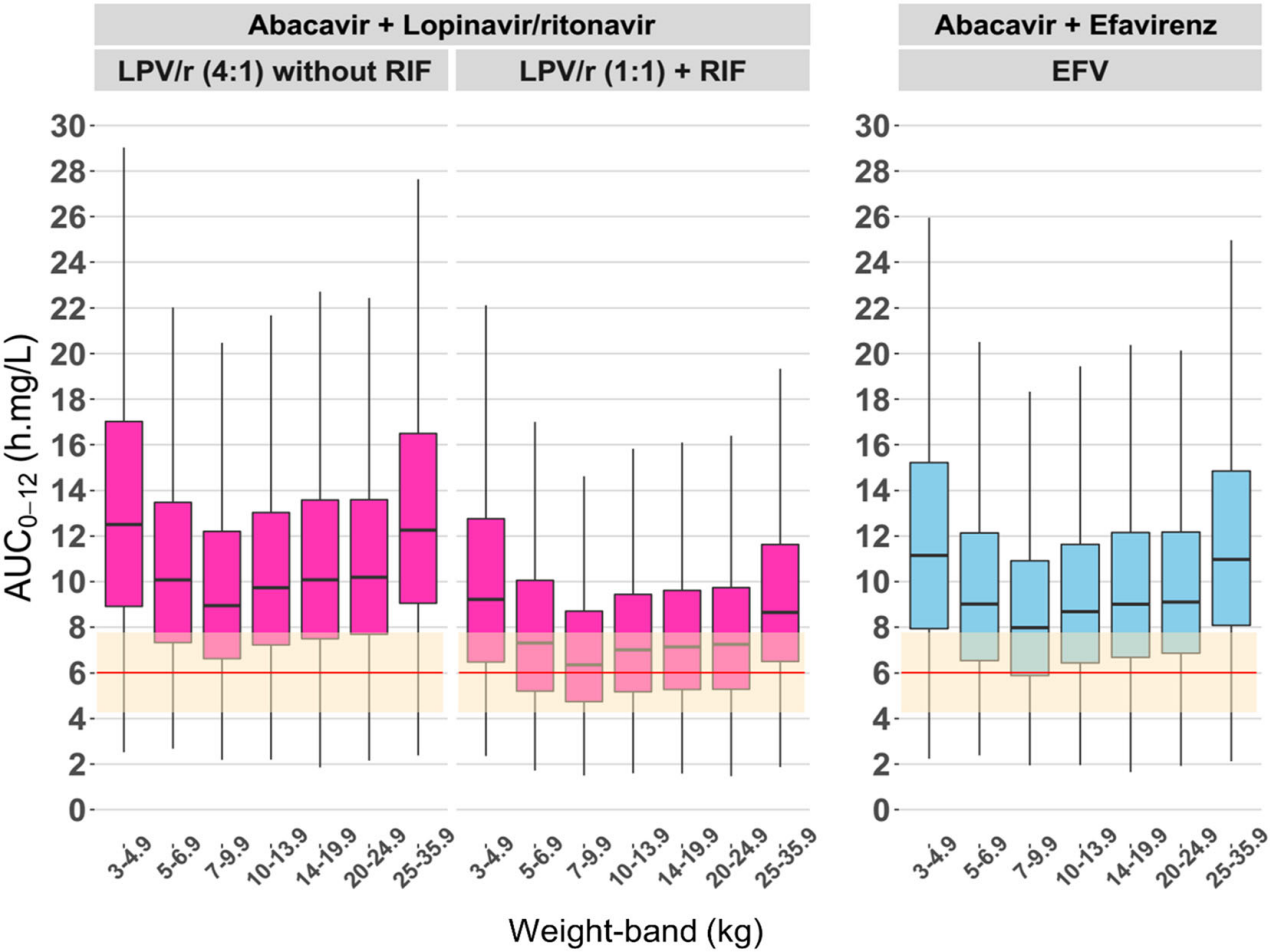
Simulated steady‐state of 8 mg/kg abacavir AUC_0–12_ *vs* body weight, by concomitant antiretrovirals with or without TB treatment. The left panel shows exposures during co‐treatment with standard LPV/r (4:1). The middle panel shows exposures in children on super‐boosted lopinavir during rifampicin (RIF)‐based TB treatment, while the right panel shows exposures in children on efavirenz (EFV). The box indicates interquartile range, while the whiskers denote the 2.5th and 97.5th percentiles. The red horizontal red line represents the recommended median adult exposure (6.02 mg·h/L)

Simulated abacavir AUC_0–12_ based on the WHO weight‐band dosing recommendations were compared to the adult recommended AUC_0–12_ of 6.02 mg·h/L, shown in Figure 4. With coadministration of LPV/r (4:1) or efavirenz, abacavir AUC_0–12_ was higher than the recommended adult AUC_0–12_, while the values were within the adult AUC_0–12_ range when abacavir was coadministered with super‐boosted lopinavir and rifampicin‐based TB treatment. Higher exposures were observed in the 3.0–4.9 kg weight group, likely due to incomplete maturation of clearance. Similarly, the heavier children in the 25–35.9 kg group receiving the adult dosage also achieved higher exposures. In contrast, low exposures were seen in the 7–10 kg weight group.

## 4 DISCUSSION

A pooled individual participant data population analysis was performed to describe abacavir pharmacokinetics in children and characterized the effect of body size, organ maturation, malnutrition and concomitant medications. The pooling of data from different studies allowed us to re‐evaluate and characterize drug–drug interactions and other covariate effects on abacavir exposure more robustly and reliably than in any single study. Allometric scaling with total body weight explained the effect of body size on the disposition parameters, while a sigmoidal function of age captured the effect of developmental change and organ maturation in the younger children. The maturation estimates were in line with previous reports,^16^, ^17^ with this effect reaching near maturity before 2 years of age, as shown in Figure 1. Since only few children below 3 months were present in our cohort, the maturation results in this age group and the predicted values for neonates have limited precision and are mostly based on extrapolation. Confirmatory studies focused on smaller children are needed.

Coadministration of abacavir with LPV/r (4:1) in the absence of rifampicin was shown by Waters et al. to reduce abacavir exposure by 30% in adults.^14^ In our analysis, we observed low abacavir clearance in children on the first day of treatment, resulting in 22% increased exposures. This could be explained by the absence of ART‐driven induction, which is generally attained within 1–4 weeks.^32^, ^33^ Even though most children were severely malnourished during the first day of treatment, the size of this effect is lower than that described by Waters et al., but they compared abacavir as a single drug against abacavir coadministered with LPV/r (4:1) at steady state, while in our analysis, abacavir and LPV/r (4:1) were administered together from the first day of treatment. The induction effect of efavirenz was 12% stronger than LPV/r (4:1) at steady state; efavirenz is a known inducer of UGT.^34^

A 29.4% decrease in abacavir exposure was identified in children treated with rifampicin‐based anti‐TB treatment and super‐boosted lopinavir. Ritonavir and rifampicin both upregulate the pregnane X receptor (PXR), which induces several Phase II enzymes including UGT, a primary enzyme involved in abacavir metabolism.^35^, ^36^, ^37^ It is uncertain to which extent rifampicin, ritonavir and/or lopinavir contributed to the effect. We previously reported that the lopinavir concentrations were similar during anti‐TB treatment and super‐boosting,^20^ likely excluding a lopinavir contribution. Moreover, when we attempted to correlate the individual values of ritonavir exposure to the decrease in abacavir concentrations, the model fit was worse when ritonavir was used instead of the categorical effect encompassing anti‐TB treatment and super‐boosting. Therefore, the estimated decrease is mostly due to rifampicin. We expected a similar effect in children on efavirenz and rifampicin. However, this effect could not be confirmed, as only three children received this treatment combination. Importantly, we expect higher abacavir exposure when coadministered with drugs such as dolutegravir that have lower potential for drug interactions.

Malnourished children experienced higher abacavir exposure, which was best described in the model with an apparent increase in bioavailability and decrease in clearance. The reason for the higher exposure may be the decreased functionality of metabolizing enzymes or altered protein levels. Indeed, total protein levels on the first day of treatment (65.0 [56.8–73] g/dL) were lower than after 14 days of treatment (77 [64–84] g/dL).^13^ At the same time, malnutrition alters the functionalities of many body systems, making it difficult to identify all factors impacting abacavir pharmacokinetics. It is worth mentioning that although malnutrition does affect plasma protein composition, its impact is very minimal compared to its combined effect with inflammation.^38^, ^39^, ^40^ Subsequently, inflammation is also associated with decreased hepatic expression of drug‐metabolizing enzymes such as CYP and UGT enzymes.^41^, ^42^ Introduction of ART is linked with reduction of inflammation and improvement of malnutrition. The effect of malnutrition on abacavir pharmacokinetics appears to recover faster than children's weight gain. This is evident in the MATCH study, where PK between visits 1 and 2 was different while there was a small improvement in weight‐for‐age *Z*‐score; see Table 1. This may explain the lack of association between malnutrition and PK in the DNDi study of children co‐treated with rifampicin where, although some patients were malnourished, the first study visit was at least 1 month after treatment initiation. Despite including all the above‐mentioned covariates, high variability in bioavailability and clearance was still observed in the MATCH study compared to the other studies, possibly reflecting the variability in the severity of malnutrition within the cohort.

In our analysis, abacavir, when formulated in fixed‐dose combination tablets with lamivudine (and coadministered with efavirenz), had slower absorption than the liquid formulation, which was coadministered with LPV/r (4:1). This is consistent with prior reports that associated the liquid formulation with an 11% higher *C*_max_ than the tablet formulation, although the difference was deemed as clinically unimportant.^9^ In all the studies in this analysis, food was given at least 2 hours after dose administration, making food an unlikely cause of the observed difference.

The observed abacavir pre‐dose concentrations (mostly 12 hours after the previous evening's self‐reported time of dose) were often higher than the concentrations observed at 8–12 hours after observed dose intake. This could possibly be due to the night dose being given later than documented, slower absorption due to coadministration with food, or diurnal variation.

In Table 3, we summarize published abacavir pharmacokinetic analyses, for comparison with our results. For each study, we included details on the study population and the dose received, the reported values of AUC and clearance. We also use allometry and the median value of body weight in each population to apply allometric scaling to clearance and allow for easier comparison of AUCs across studies. In general, AUC_0–12_ of abacavir during coadministration with efavirenz or lopinavir/ritonavir with or without rifampicin was comparable to the adult target, as well as exposures seen in other studies conducted in children, the exception being in the severely malnourished children whose exposures were variable and higher compared to their counterparts.

Our analysis suffers from limitations arising from data pooling from diverse studies. These include unequal distribution of covariates between studies, such as first‐dose abacavir concentrations only being available in the dataset of malnourished children, and more frequent use of liquid formulations in younger children and with lopinavir/ritonavir. Also, although the abacavir assay was performed by different laboratories, both laboratories participate in international quality assurance and proficiency testing schemes and should have comparable standards, with systematic differences between assays addressed in the PK model. We believe that these challenges have been well handled in our analysis with the use of nonlinear mixed‐effects modelling which was specifically developed to account for the concomitant effect of multiple factors.

To conclude, the findings from this pooled analysis present robust abacavir parameter estimates and characterization of the effect of relevant covariates such as body size, age and concomitant medications, since the results are based on a large number of participants from different settings. There was a decrease in abacavir exposure from first dose to steady state when coadministered with LPV/r, being even more pronounced when coadministered with efavirenz. This analysis confirmed our earlier finding that children with TB on super‐boosted LPV/r plus rifampicin had reduced abacavir exposures but the exposures were still similar to those reported in adults. On the contrary, malnourished children had high and variable exposures, but the exposure normalized as the nutritional status resolved. Abacavir concentrations should, however, be confirmed in TB/HIV coinfected children on EFV‐based ART with rifampicin‐based TB treatment.

## COMPETING INTERESTS

The authors have no conflicts of interest to declare.

## CONTRIBUTORS

T.T. wrote the manuscript; T.T. and P.D. analysed the data; H.M., D.B., D.G. H.R., J. L, M.L., M.C., M.A., S.H. and P.D. designed and performed the research; all authors contributed to the review of the manuscript and approved the final version for submission.

## Data Availability

The data that support the findings of this study are available on request from the corresponding author. The data are not publicly available due to privacy or ethical restrictions.
